# 

**Published:** 1912-09-15

**Authors:** 


### ANNOUNCEMENTS.

Pneumatic and Electrical appliances are such an important feature in all dental equipment that we feel sure our subscribers will be interested in the catalog of equipment advertised in this issue by the ELECTRO DENTAL MANUFACTURING CO. of Philadelphia.

In place of the usual bare description and price list, this catalog goes a great deal into the use of the apparatus and the reason for following out certain lines of construction. This ought to prove very interesting to all those interested in dental work.

A copy of the catalog may be had by addressing the company direct.

